# Beliefs and perception of ill-health causation: a socio-cultural qualitative study in rural North-Eastern Ethiopia

**DOI:** 10.1186/s12889-017-4052-y

**Published:** 2017-01-26

**Authors:** Mesfin H. Kahissay, Teferi G. Fenta, Heather Boon

**Affiliations:** 1 Department of Pharmaceutics and Social Pharmacy, School of Pharmacy, College of Health Sciences, Addis Ababa University, P.O. Box 1176, Addis Ababa, Ethiopia; 2 Leslie Dan Faculty of Pharmacy, University of Toronto, 144 College Street, Toronto, ON M5S 3M2 Canada

**Keywords:** Health, Illness, Beliefs, Indigenous, Spirit, Ethiopia, Qualitative

## Abstract

**Background:**

Understanding perceptions of the causes of ill-health common in indigenous communities may help policy makers to design effective integrated primary health care strategies to serve these communities. This study explored the indigenous beliefs of ill-health causation among those living in the *Tehuledere* Woreda /district/ in North East Ethiopia from a socio-cultural perspective.

**Methods:**

The study employed a qualitative ethnographic method informed by Murdock’s Theory of Illness. Participatory observation, over a total of 5 months during the span of one year, was supplemented by focus group discussions (*n* = 96 participants in 10 groups) and in-depth interviews (*n* = 20) conducted with key informants. Data were analyzed thematically using narrative strategies.

**Results:**

In these communities, illness is perceived to have supernatural (e.g., almighty God/ Allah, nature spirits, and human agents of the supernatural), natural (e.g., environmental sanitation and personal hygiene, poverty, biological and psychological factors) and societal causes (e.g., social trust, experiences of family support and harmony; and violation of social taboos). Therefore, the explanatory model of illness causation in this community was very similar to that of the Murdock model with one key difference: social elements need to be added to the model.

**Conclusion:**

Members of the study community believes that supernatural, natural and social elements are linked to ill-health causation. A successful integrated primary health care strategy should include strategies for supporting patients’ needs in all three of these domains.

**Electronic supplementary material:**

The online version of this article (doi:10.1186/s12889-017-4052-y) contains supplementary material, which is available to authorized users.

## Background

Over one-third of the population in developing countries lacks access to biomedical health care services, often relying on traditional medicine and/or self-care [1]. In addition to their physical inaccessibility, biomedical health services are often unaffordable. Indigenous people often believe that Western trained doctors are not equipped to address their concerns which are can include spiritual as well as physical concerns [2]. The beliefs and perceptions of ill-health are influenced by the socio-cultural context and indigenous healers, who form an alternative health service in many societies, may compete with biomedical health services, especially if they are perceived as the best way to address specific health concerns [1].

Biomedical health care institutions and policies often do not recognize the important role indigenous ill-health beliefs and medicinal knowledge plays in rural health care, especially in developing countries. Too often, indigenous medicine is criticized as “harmful traditions” as if it was a threat to human health. However, others acknowledge that indigenous medicine should be embraced as an important part of health care systems in indigenous communities [3–5].

In Ethiopia, people use indigenous medical systems as an alternative health care service along with the biomedical health services [1, 6]. Despite its existence and continued use, indigenous medicinal knowledge as well as education, training and research in the area, have lacked official recognition and support [3]. This study aimed to explore the socio-cultural context of indigenous beliefs regarding ill-health among five *Tehuledere* communities found in the Amhara Regional State of Ethiopia. *Tehuledere* was selected for the study because of the rich and unique indigenous medicinal beliefs, and practices in the area.

### The context: *Tehuledere* region

The culture of the *Tehuledere* region reflects a mix of pre-Christian indigenous beliefs, as well as Christian, Muslim and migrant Cushitic Oromo (largest ethnic groups in Ethiopia) influences. After their migration from the southern part of Ethiopia, before embracing Islam as their religion, the people of the *Tehuledere* region continued to make use of their indigenous Cushitic beliefs and practices [7]. The indigenous religion of the people of this region recognizes the existence of a Supreme Being and other lesser spirits, namely, the ‘*ayana’* spirits which are believed to serve as intermediaries between man and the Supreme Being [8, 9]. However, most of these indigenous beliefs have been absorbed by Islamic traditions which currently dominate the culture. Our study highlights the importance of understanding the perceptions of causes of ill health and disease in the study communities as a guide to how best to implement public health initiatives in the region.

### Theoretical framework

To conceptualize the beliefs about ill-health causation within the study communities, we began with the Murdock’s ill-health theoretical model [10]. According to this theory, it is important to distinguish between beliefs about the natural and supernatural causes of illness and disease. In this framework, natural causes include beliefs that the impairment of health is a physiological consequence of some experience of the victim that is consistent with Western biomedicine. This broad category includes five distinct types of natural causes of illness: infection, stress, organic deterioration, accident, and overt human aggression. According to Murdock, supernatural causes include: theories of mystical causation (i.e., fate, ominous sensation, contagion, and mystical retribution); theories of animistic causation (i.e., soul loss, and spirit aggression); and theories of magical causation (i.e., sorcery and witchcraft) [10]. Other studies suggest that different societies give varying degrees of importance to theories of natural compared to supernatural causes of illness [11–13]. This dichotomy was a key sensitizing concept that guided the design of this study.

## Methods

### Design and setting

**Fig. 1:**
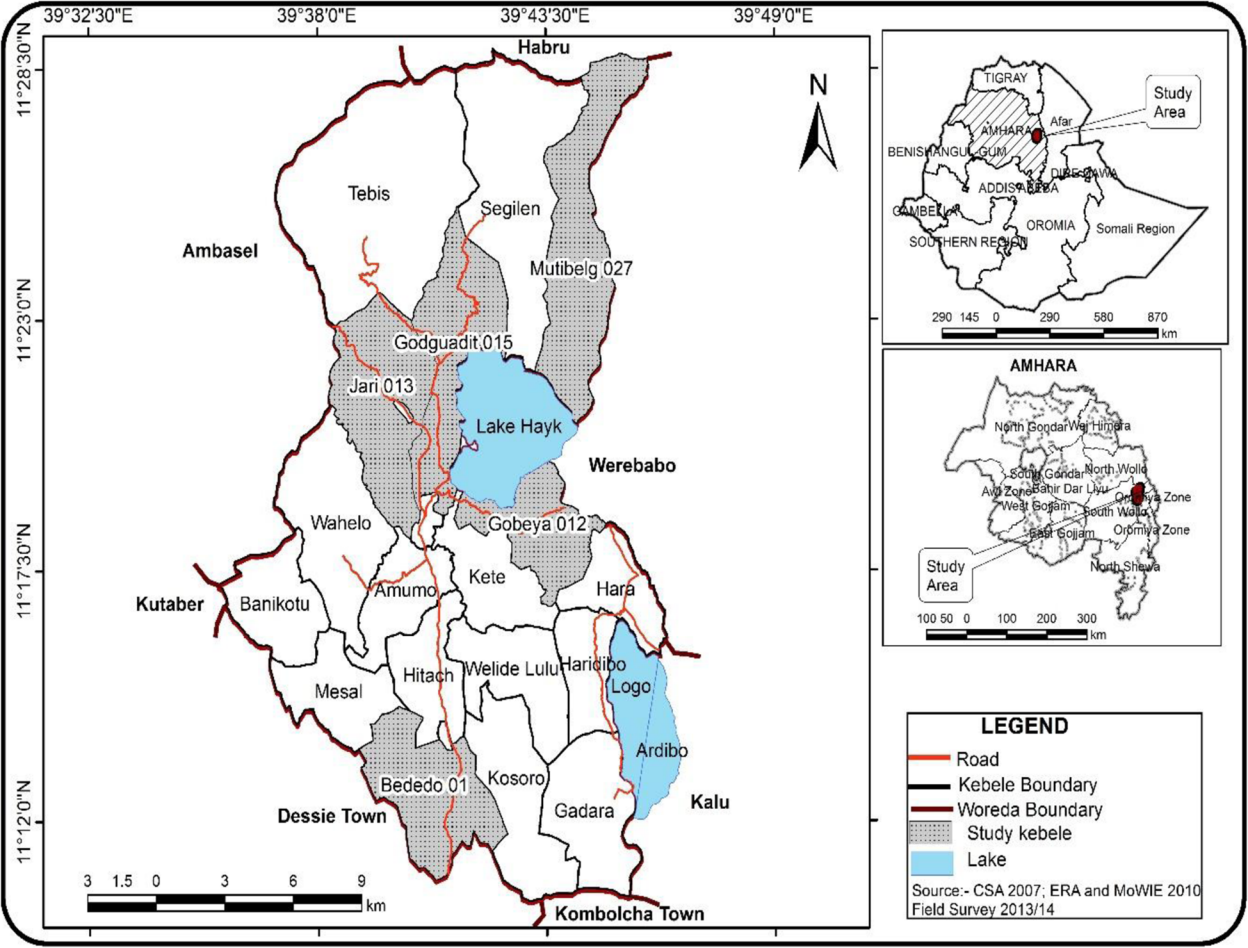
Map of *Tehuledere* Woreda, South Wollo Zone, Amhara Regional State, North-eastern Ethiopia, 2014 [15]

A qualitative ethnographic method was conducted in *Tehuledere Woreda*, an administrative unit in the north-east of Ethiopia [14] (see Fig. 1). The capital of the *Woreda*, Haik, is situated 430 kms away from Addis Ababa, the capital of Ethiopia. According to the *Tehuledere Woreda* Information Office (TWIO), the *Woreda* covers an area of 45,800 ha with a population of 152,107. There are 23 *Kebeles* (the smallest local administrative unit) administered by the *Woreda*, including 19 rural, 2 urban and 2 semi-urban towns. Five rural communities (one from each of the five *Kebeles*) were included in the study. The people in this area are members of the Amhara ethnic group and are largely Muslims. They speak Amharic, Ethiopia’s official language. As predominantly rural *Woreda,* most inhabitants rely on farming. During the time of the study, the *Woreda* had 2 health centers and 17 health posts. In 2014, communicable diseases including malaria, lung infections, diarrheal, intestinal parasites, eye infections, skin disease, and rheumatism were the major public health problems in the area [15].

### Sampling procedures and accessing the field: getting in

Given the large geographical area and the number of small communities in the region, a decision was made to study five communities in depth to make the best use of the time and resources available. The investigators consulted the *Woreda* health extension workers (primary health care practitioners) on the selection of study communities. The accessibility and geographic distribution of the five *Tehuledere* sub-districts (‘*ketena’*) around the urban center provided the primary basis for selecting the study communities. The other criterion was the interest shown by the health extension workers to collaborate in the study. The five study communities, selected were: *Gobeya, Godguadit, Bededo, Jari and Muti-Belg.* The health extension workers also helped to select 96 participants for 10 focus group discussions and 20 individuals (two from each focus groups) were selected purposively for follow-up individual interviews based on their demonstrated knowledge and ability to describe their experiences in the focus groups. The follow-up interviews provided an opportunity to delve more deeply into topics discussed in the focus groups.

### Data collection and analysis

This ethnographic study was grounded in participant observations made by the principal investigator (MHK) working as a volunteer heath extension worker in the study area for five months between June and November, 2013. This volunteer participatory observation helped him to access research participants and health extension workers working in health institutions. The researcher was also able to make substantial observations of local activities such as rituals, festivities, public gatherings involving health practices, and converse informally with community members. Data obtained from observations and conversations were recorded in field notes, photographs, and audio-recordings.

Following introductory discussions with community leaders/representatives, ten focus group discussion sessions (one all male and one all-female in each *kebele*) were conducted. Study participants were adults over 30 years of age identified by health extension workers in the area to be knowledgeable about local health traditions who were willing to participate in a conversation about their health. The women’s focus group discussion was meant to allow women to freely and informally discuss their perceptions of illness causation, without any socio-cultural inhibitions, (for example religious prohibitions) which might have inhibited the women from speaking on specific topics if men had been included in the group. The focus groups lasted 1.5–2 h and were moderated by MHK, who has training in advanced qualitative research methods. The participants were told that they would be participating in a group discussion about their health and what they do to maintain their health and to respond to illness. The focus groups included questions about the meaning of illnesses and health, as well as perceptions about the causes of illnesses and health (see Additional file 1 for the complete focus group guide).

Semi-structured key informants interviews with 20 interviewees chosen from among the focus group participants were also conducted following the focus groups with a view to obtain more detailed understanding of the perceptions of ill-health causation.. Interviews lasted 1–1.25 h, were conducted in the informants’ private homes or while sitting in public spaces in the villages (See Additional file 1 for the complete interview guide). Focus groups and interviews continued until the data in the key emerging themes were saturated (i.e., key points were repeated and no significant new information was emerging) [16, 17].

All focus group discussions and interviews were conducted, audio-recorded and transcribed verbatim in Amharic. Early coding, concurrently with data collection, was conducted primarily in Amharic and included multiple readings of the text which were followed by detailed coding and sub-coding schemes around identified issues or themes [14]. Two analysts (MHK and another Amharic speaking team member, TS) immersed themselves in the data by reading and open coding the transcripts independently and developing preliminary codes. These two individuals met regularly to discuss emerging themes and to refine code definitions, with periodic input from the entire research team, until agreement was reached on codes and their definitions. . Each transcript was coded line by line and these codes were organized into higher-order conceptual themes. Sections of original transcripts and key quotes considered to be illustrative of the emerging themes were translated into English to facilitate discussion with the full research team as needed, because one of the research team members, HB, was a non-Amharic speakerIndividual codes and themes were discussed at group meetings until consensus was reached on basic themes and subthemes across the focus groups and interviews. Finally the themes were incorporated into a conceptual model of the participants and their beliefs and perceptions in illness causation [18].

All qualitative data from participant observations, in-depth interviews, fieldwork, personal memos and informal conversations were organized using NVivo 10 computer software.

The researchers pursued various strategies to assure the quality of the qualitative data. For example, the research findings were shared with research participants and the local research assistants who confirmed the interpretations accurately reflected their perceptions and experiences. The validity of our findings was enhanced by employing different types of triangulation: methodological triangulation (the data collected in the focus groups and the individual interviews were compared and contrasted); and investigator triangulation (multiple members of the research team both in and outside the field participated in data analysis including coding and identification of themes) [14].

Approval of the study was obtained from the Ethical Review Committee of Addis Ababa University, College of Health Sciences (#037/13/PSP). Permission to conduct the participant observation in the communities was provided by the heads of the health facilities. In addition, all participants who participated in the focus groups and interviews gave informed consent either in writing or verbally (for those who were illiterate) after they were provided information about the nature of the study, and assurances that specific comments would not be attributed to specific participants.

### Issues of reflexivity: MHK status as an indigenous ethnographer

The first author’s (MHK) “native” status offered both opportunities and limitations for the study [19]. He approached this ethnographic work as an “Amharic” speaker and tradition bearer, a member of the “Amhara” elite, and also as a senior pharmacy professional. He was able to use existing networks and contacts within the indigenous institutions, including traditional leaders and local health officials, thereby gaining access to a very wide cross-section of people. He carefully reflected on how the data collection process influenced his own perceptions, and how other people respond to him. He was also faced with the challenge of being perceived as a powerful individual due to his position as a member of the elite and a senior university lecturer. The use of open-ended questions, as well as informal conversations with informants on topics they themselves raised, were among the ways pursued to mitigate these challenges.

## Results

### Demographic characteristics of participants

In total, 96 people participated in focus groups. The number of participants in each focus group ranged from 8 to 12. Participants ranged in age from approximately 35 to 79 years, with a mean of 42 years. Most participants were Muslims (*n* = 92) and the rest were Ethiopian Orthodox Christians. Most of the participants were married. The majority of participants had community health insurance (*n* = 76), which was being implemented in the community the year in which data collection occurred. More than half (*n* = 53) reported that they were illiterate, i.e., didn’t read and write Amharic. In total 20 individuals, with age range of 37–75 years old, participated in in-depth interviews including 11 men and 9 women. They were very similar to the focus group participants in their demographic characteristics.

### Beliefs and perception of ill-health causation: a conceptual understanding

We found that the “causes” of ill-health were constructed and negotiated within the socio-cultural context of the study communities. The narratives of health among *Tehuledere* communities include three major themes that explained causes of ill-health: (i) supernatural (ii) natural elements or physical causes: (iii) social elements such as mistrust, social support/family dynamics as well as violation of taboos and moral injunctions. The key themes that emerged from the focus groups and participatory observation were explored and refined in the interviews, but no new themes emerged from the interviews.

### Supernatural causes of ill-health

Similar to Murdock, a key category of causes of ill-health was the supernatural described by members of the study community as forces capable of putting spells on human beings. These forces also have the ability to heal one from these spells. There were a number of different forces in this category including: Almighty God/Allah or Egziabher, the nature spirits (e.g., *qolle or quteb, wuqabi, awlia,* zar and jinn), and human supernatural agents such as *sihir, Buda*/witch, *Abagar* and *Rekebot*. Each of these sub-categories will be described briefly below.

#### God/Allah

Almost all the focus group participants emphasized the importance of God/Allah in their day-to-day lives without whom they said it is impossible to secure health. Health was attributed by study participants to the will of God/Allah Who sends ‘*Melayka’* (the guardian angel) into people’s homes:

*Many thanks to Allah!* … *If Allah wants you to stay healthy, you stay healthy. I have never gone to modern health centers. At home, we spread grass on the floor, we chew “Khat” in the name of ‘Abduye’, ‘Kedir’ or ‘Nura Hussien’ [the gods for Wednesday, Saturday and Tuesday respectively] to get the “Melayka” (angel) into our homes, to get the ‘Wukabe’[nature-spirit] close to us, to be heard by the gods. Then we stay* healthy *[Female, Study Community # 4].*

*Being healthy means “alemelekef” [not having a spell]. Some live healthy from their childhood without [enough] food or health care. If God wants you to live healthy, then you live healthy [Female, Study Community # 1].*

Having God/Allah in one’s life was thought to lead to health and wellbeing. Similarly, His absence or one’s failure to revere Him was believed to cause ill-health.

#### Nature spirits

The study participants attribute many ailments to withdrawal of nature spirits’^1^ (e.g., *qolle or quteb, wuqabi, awlia,* zar and jinn) protection and wrath. These forces are believed to have unlimited power. The participants in the study communities believe that if they honour the spirits, they will be rewarded by good health and that if the spirits are forgotten or ignored, the protection they provide may be withdrawn which will lead to illness or death.

For example, *Wadaja*, a communal prayer ceremony, is a common rite practiced in *Tehuluedere*. It involves praising and glorifying the sky-god and seeking his spiritual assistance to ward off evil. A male participant discussed the relevance of *Wadaja* for health:

*Wadaja is held particularly when some potential or actual problem which would affect the whole community, some village members, a family or an individual is imminent. Wadaja lately assumed a much more modified and purposive role of combating the zar, buda or other supposedly spirit afflicted illnesses [Male, Study Community # 2*]*.*

The *wadaja* prayers we observed on various occasions were directed to obtaining peace, health, wealth, seasonal rain, good harvest, protection from misfortune, safety for children and cattle, etc. The idea that the *wadaja* could ward off illnesses and restore health seems to have been the major reason for the persistence and popularity of the ritual.

A second well respected ritual spirit, articulated in all five female focus groups as a spirit for health, is ‘*Chelle*.’ Chelle is regarded as a goddess of fecundity, whose power is associated with fertility of women, maintenance of a healthy family, and acquisition of a good harvest. Chelle is represented by colorful small beads.

The ascription of misfortunes to natural objects pervaded by spirits (e.g., *qolle* or *quteb*) was observed to be common in the study population. Sicknesses, epidemics, contagious diseases and even death were explained in terms of punishment by enraged *qolle* spirits. Qolle spirits were also described as guardians who exact tributes in return for physical and emotional security (including health) and who deal out punishments for failure to recognize the spirits:

*I believe in qolle spirits or ‘adbars’. …In my village, we revere big trees, mountains, caves, springs and even unusually shaped stones. Qolle spirits are mostly venerated by buttering the natural objects, burning incense, tying around these objects strips of ropes or even a bunch of hair [Female, Study Community # 5].*

*In our tradition people believe in qolle spirits (the spirit of individuals) is a guardian from illness and cause of illness. To act in this capacity qolle must be acknowledged and shown respect through appropriate rituals such as scarification of goats, respecting the local customs and values, etc., the spirit of the qolle are good for keeping relatives safe from harm and maintain health [Male, Study Community # 3].*

#### Human supernatural agents causing ill-health

Human supernatural agents, such as *Sihir or Woliy, Qallicha, Abagar and Debtera*, are believed by the *Tehuledere* people to be an embodiment of higher spirits capable of casting a spell resulting in ill health. In other words, the spirits supposedly reside in some human beings and rituals and/or prayers need to be performed to withdraw the evil spirits impacting ordinary people through these human agents.

For example, the *qallicha* 2 witches are believed to cast spells on people causing them to experience ill health:

*The sheikhs and qallichas apply magical knowledge to affect cure or induce illness…… Muslim sheikhs consult medical texts, produce magically charged protective amulets (Kitab) and talismans (talsam) of various kinds believed to endow the bearer with some beneficial effect [Male, Study Community # 4].*

Another example is the initiation of men by the *Abegar,* a name given to male community opinion leaders who are regarded as human agents who invoke the cult as the instigator of ill-health. Study participants explained that they believe that if a person violates the required norms, his/her health will be in danger:

*If a person makes an oath in front of the Abegar with his hands on the Rekebot /an indigenous spot made of mud soil in which isen is served or place of truth/,and then tell lies, he will face problems. Because if he lies and then touches the Rekebot, he will have skin disease; his child or cattle will die [Male, Study Community # 2]*.

### Natural causes of ill-health

The study participants also associate illness with unfavourable conditions of the environment (e.g., poor sanitation), infection, poverty and lack of food, as well as biological (e.g., aging, genetics) and psychological causes such as stress and worry. Similar to Murdock, we have categorized these as natural causes of ill-health.

#### Environmental sanitation, personal hygiene and prevention of infection

Environmental sanitation is one key cause of illnesses that was recognized by all participants:

*The water around the lake has altered its nature. Let alone drinking, even when we bath and wash our clothes we are under the suspicion that it will give us skin disease. When we drink it, we contract worms, they twirl in the stomach and give our kids ‘tik, tik’ [Stomach- ache, salivate and followed by vomiting,] [Male, Study Community # 5]*.

Infections were also identified as a common cause of illnesses:

*The use of non-sterilized sharp things for removing a child’s milk teeth and female circumcision is consequential. People in our village sterilize the equipment they use for these purposes. Many become sick from the filth on the sharp equipment [Male*, *Study Community # 4].*

#### Poverty and lack of food as causes of ill-health

Many of the participants in the study specifically related poverty and lack of appropriate food to ill health. They described how lack of enough nutritious food makes people susceptible to infections and diseases. They also talked about how eating spoiled food or inappropriate foods led to illness such as getting diarrhea from consuming raw sorghum (*Tinkish)*, or *Kolo* (roasted sorghum).

Almost all the focus group participants identified better food production as a contributor to health:

*We keep healthy when we produce enough food and have work to do. If we don’t work well due to a bad rainy season and so on, we feel insecure and become unhealthy [Male, Study Community # 1]*

Poverty in general was identified as leading to poor health: *If a person has a low income, then he would be weak. He would not be able to have enough food, this affects his health…We’ve become subject to ‘Ke ijje Wode Afe Nuro’ (hand-to-mouth livelihood) [Male, Study Community # 3].*

#### Biological and psychological factors

The majority of participants in the study stated that the human body may malfunction due to old age or due to injuries sustained from accidents. Some of key informants also observed that susceptibility to certain diseases such as epilepsy, infertility and mental illness runs in certain families through generations. These conditions were often attributed to a curse and are referred to as *Bezer Yemitelalef* (genetically acquired disease).

Descriptions of psychological causes of disease were also quite common in these communities:

*Well, if there are no hustles at one’s home and it’s peaceful, the person becomes healthy. So if he is able to work well and come back home without any stress, he becomes healthy [Male, Study Community # 1].*

Presence of worry was also seen as part of being cause of illnesses, as one female participant explained:

*If he is angry, then he won’t do his work, he would just be distracted by worrying too much, I think that might lead to other diseases….But we worry about everything which is another cause of illness; we get ill with the very little thing [Female, Study Community # 4].*

### Social causes of ill-health

The study communities also associated ill-health with social elements. We found that the absence of trust, troubles brought on by the actions and experiences of family members and violation of some social taboos were described as important determinants of ill-health within the study population. This theme does not appear in Murdock’s model.

#### Trust/Lying

For the study communities, a culture of social trust was central to their identity, and they believed this was directly connected to the health of the body. For example, if someone violated the trust of a significant other or another member of the community, they believed the consequence would be illnesses. Participants in most focus groups maintained that trust in one’s married partner determines one’s health. For example, if a man cheats on his wife, this could cause illnesses:

*A person will be healthy when he settles at home, and is not cheating on his wife. A settled man lives in peace and prevents himself from syphilis and gonorrhoea. [Female, Study Community # 2]*

Many respondents identified lying as a cause of ill-health. For example, there are beliefs that health problems and/or an illness or death will occur if one gives false witness in front of *Jamea* (a group of community opinion leaders, usually Muslim clergy)*.*

*… If you don’t tell the truth, you and your family will fall ill. The disease is said to pass onto seven generations. [Female, Study Community # 5]*

#### Social support/Family dynamics

Stress from family dynamics or lack of social support from families were also identified as being potential causes of illness:

*…if I don’t discuss good things with my husband and kids, if we’re unhappy, there will be neither peace nor health in the family [Female, Study Community # 1].*

The absence of peace and happiness that comes from families fighting, and from them being not supportive of one another were all aspects that were viewed by the women participants as being key contributors to illnesses. In contrast a stable home and family was seen as an important contributor to health.

*We say in our culture, if there are arguments and fights there will be no peace then, it would shorten your life and laughter will be away from you. So if there is no peace at home, there will be no health [Female, Study Community # 4].*

*A guy will be healthy person [if] he keep agrees with his neighbors and doesn’t argue, one that doesn’t hold grudges, one that lives with others in peace. [Female, Study Community # 3].*

#### Violation of social taboos

We also found acts in violation of social taboos or moral injunction were thought to cause illness directly rather than through the mediation of a supernatural being. An example cited in most of the focus groups was that of disrespecting elders, *Abegar* or Sheikh and a link to how that causes illnesses:

*If people anger the Abegar or sheik, they get hurt by the ones they offended. I saw it happen as a child. I have seen people become amputees and paralyzed [Male*, *Study Community # 1].*

*Disobeying elders causes ailment. Not listening to them means we are exacerbating the disease we have [Female, Study Community # 2].*

Violation of time ban taboos were also said to contribute to illness:

*Leaving, travelling in the wrong time will cause disease from attack by the devil (lekeft). We consider people who travel at this wrong time bad, harmful, killers and thieves or people into something tricky. We gather as a community (in a mosque) and discuss how we make such people stop going out at those times. If we see someone leave home at the wrong time, we say “he is going to bring affliction” [Male, Study Community # 4].*

## Discussion

This study found that among the *Tehuledere* there are a wide range of perceptions regarding the causation of illness. As noted in other studies, effective primary care requires that all health care providers and policy makers take culture and traditional into account [20]. Similar to other cultural groups in developing countries around the world [11], many of the *Tehuledere* people attribute illness to the wrath of supernatural forces. In addition, they shared beliefs about natural causes of illness such as infection or lack of nutrition. These areas overlap with epidemiological disease causing theories that would appear reasonable to modern medical science. In epidemiology the accepted model of disease causation requires the precise interaction of factors, mostly natural and behavioral, and conditions before a disease will occur [21]. A more interesting finding was that they also articulated how important social relationships are in maintaining good health. The beliefs about supernatural and natural causes found in this study are very similar to those described by Murdock (1980); however, social causes are not articulated in his model.

### The importance of the supernatural

Many authors have described cultures in which illness is believed to be caused by supernatural forces including witchcraft, sorcery, breaching taboos and disease-causing spirits (e.g., [22–25]). Supernatural explanations also dominated the discourse of ill-health causation in our study. Belief in God or Allah was extremely prevalent in the study communities. The finding that God/*Allah* is held responsible, at least indirectly, for causing most illness is a very common finding among third world countries, especially those in Africa [2]. For example, Lidell and his colleagues (2005) described how Sub-Saharan people’s relationship with God takes a form of nature-spirit reverence and connection which is vital to maintaining family health [26]. Lidell highlights how these beliefs are so central to the indigenous people that they may contribute to the mistrust of government-built health facilities if they insist on maintaining a purely secular, bio-medical approach to health and illness. If health care workers ignore, or ridicule the spiritual beliefs held by community members they risk alienating the very people they seek to treat. For the followers of indigenous faith, one’s health status is not balanced without the nature-spirit connection. Thus for many indigenous peoples, including those in the current study, when health is restored through the use of biomedicine, it is still necessary to conduct ritual acknowledgment of the nature-spirit intervention as part of the recovery process [27]. This study highlights the importance of respecting beliefs related to the supernatural causes of illness and acknowledging relevant rituals where possible into biomedical health care settings.

### Social relations as a cause of ill health

Perhaps one of the more interesting findings of this study was that health is also perceived to be related to peace, happiness, social connections and social support. The study communities rejoiced in their culture of eating together and sharing with those in need. The link between lying and ill health appears to be unique in this culture. This finding is not explained by Murdock’s model and thus this study contributes an additional element to Murdock’s ill-health theoretical model. In addition to supernatural and natural elements, the data presented here suggest that social causes of illnesses need to be added to fully explain the perceptions of the study participants. Data obtained from our study indicated that the role of social prohibitions and the consequences of their violation were a key factor causing health problems in *Tehuledere*. The connection between ill-health and lying needs further exploration for indigenous groups like this.

### Implications

Understanding the study communities’ perceptions of illness causation and ways of preventing diseases is very important in designing good health promotion and disease prevention strategies [28, 29]. The respondents in the present study perceived that supernatural forces were the most important cause of ill health. They also articulated the role of natural causes and social relationships in maintaining good health. Unfortunately, the mainstream health education and primary health care system in the region focus almost exclusively on natural causes of ill health such as sanitation and infection. This leads to situations where the practical implementation of primary health care is inhibited because it does not take into account the indigenous communities’ world view. When Western biomedical practitioners overlook, or scorn the supernatural convictions held by group individuals they risk distancing the very individuals they look to treat. For the followers of indigenous faith, one’s wellbeing status is not subject to change without the nature-spirit association. Consequently for many indigenous people, attempts to restore wellbeing through the utilization of biomedicine must include customary affirmation of the nature-spirit intercession as a feature of the recuperation process. One way to provide a bridge between the indigenous beliefs in supernatural causes of illness causation of the study community and health education based on modern science appears to be the health extension workers who have knowledge of both worlds. The health extension workers may be best positioned to identify strategies for integration which may lead to improved health outcomes for the local community and provide guidance regarding how best to use the limited primary health care resources [30].

The current Ethiopian indigenous health strategy (the National health strategies with regard to Indigenous Solution reported in March 2015) acknowledges the need to support components of indigenous medication including recommendations to explore potential beneficial aspects [31]. However, the capacity to execute and give expanded resources to support the sustainable utilization of indigenous medicine and implement health strategies that take into account indigenous ill-health beliefs is constrained. Issues identified with administration and bureaucratic frameworks don’t fit well with indigenous ill-health beliefs. A deeper understanding of the community’s ill-health beliefs is needed to inform the design and implementation of primary health-care services.

Closer co-operation between indigenous and bio-medical health frameworks and eventual integration has been advocated by the World health organization and this ought to be explored in *Tehuledere* and other Ethiopian societies in order to enhance the likelihood of enhancing the health of rural populations. Our study provides an excellent example of why health education and primary health care delivery interventions need to take into account what people actually do and the beliefs that drive their actions. Health education and care solutions found to be effective in one system may not work as well in other settings [32]; however, some of the lessons learned from this study may be applicable to other cultural contexts with similar beliefs. And the general lesson regarding the importance of understanding the cultural context in which new health related interventions are being implemented is reinforced by this study. It is hoped that the model of illness causation identified in this study will help to enhance the rural health plan for the 5 villages of *Tehuledere Woreda* and beyond.

### Strength and limitations of the study

Like all research, out study has both strengths and limitations. Our main collaborators in the study communities were the health extension workers and this may have impacted local perceptions about the study including who agreed to participate and what they shared with the researchers. Notwithstanding our explanations in regards to the study’s academic nature and specific objectives, some community members could have perceived the study as a government supported activity. It would seem that any apprehensiveness is likely to have been eased, by the generally friendly and mutually respectful rapport most people have with their respective health extension workers. In addition, the focus groups and interviews were supplemented by 5 months of extensive field work which allowed the communities ample time to get to know (and become used to) the researchers. This level of familiarity would have increased the comfort participants felt sharing with the researchers.

The results of this study were based on a sample of purposively selected participants who identified as individual from *Tehuledere* community. The sample was diverse in terms of age and sex and agro-ecological condition of the setting. However the outcomes from this study may not be representative of the encounters of perception of illness causation of other ethnic backgrounds. Triangulation of different data collection techniques including focus group, semi-structured interview data, observations and informal interviews with study participants was a strength of the study. Similarly, a theoretical approach initially used to frame and subsequently to interpret the data from this study allowed for a more thorough explanation of beliefs and perceptions of illness causation.

## Conclusions 

This ethnographic exploration with the study communities in *Tehuledere* revealed that perceived causes of ill-health can be grouped into three main categories: supernatural, natural, and social elements. Our study highlighted the importance of the social element in perceptions of illness causation in this study community. This finding, especially the link between lying and ill health may be relatively unique to this cultural setting. Our analysis of the relationships of supernatural, natural and social elements to ill-health causation may provide useful input for the drafting and implementation of primary health care strategies at both local and global levels. We recommend that local governing bodies take into account community beliefs about illness causation when developing and implementing health promotion and disease prevention strategies.

## Additional file

Additional file 1: Focus Group Discussions and In-depth Interviews Guide. (DOCX 17 kb)

## Footnotes

1 Nature spirit- in *Tehuledere*, is the belief in *qolle* or *quteb, wodaja*, *mewokel* and *chele* spirits that is, venerating certain natural objects as sacred and the abode of spirits. Many natural objects were believed to be capable of mediating between the “supreme deity and the ordinary humans”. The ascription of misfortunes to natural objects pervaded by spirits seems to have been prevalent where by sicknesses, epidemic or contagious diseases and even death were explained in terms of punishment by enraged spirits.

2
*Qallicha: A person known, among traditional Muslim Ethiopians, to have special spiritual power and knowledge; he is believed to be capable of forecasting the future as well as revealing hidden facts, causing illnesses and resolving individuals’ problems.*

## References

[1] Bishaw M (1990). Attitudes of modern and traditional medical practitioners toward cooperation. EMJ..

[2] WHO. “Legal status of traditional medicine and complementary/alternative medicine.” A world wide review. Geneva: World Health Organization; 2001.

[3] WHO. “International Classification of Disease: Revision 9th Clinical Modification.” 5th, Millennium edition U.S Department of Health and Human Services; 2000.

[4] Winkelman M (2007). Culture and health: applying medical anthropology.

[5] Waddel A, Petersen AR. “Just health inequality in illness, care and prevention.” Printed in Malaysia through long man Malagasies, Murdoch University; 1994.

[6] Kassaye KD, Amberbir A, Getachew B, Mussema Y (2006). A historical overview of traditional medicine practices and policy in Ethiopia. Ethiop J Health Dev.

[7] Ali A. “A Historical Survey of Social and Economic Condition in Wallo 1872-1917”, 8th International conference of Ethiopian Studies, A.A. 1984; p. 1.

[8] Trimingham S (1964). Islam in East Africa.

[9] Krapf L (1968). Travels, researches and missionary labour during an eighteen years’ residence in Eastern Africa.

[10] Murdock G (1980). Theory of illness. A world survey.

[11] Foster M, Anderson B (1978). Medical anthropology.

[12] Foster GM (1976). Disease etiologies in non-western medical systems. Am Anthropol.

[13] Young A (1983). The relevance of traditional medicinal cultures to modern primary health care. Soc Sci Med.

[14] Bryman A. Social Research Methods. 4th ed. New York: Oxford University press; 2012.

[15] TWIO. Tehuledere Woreda Information and Communication Office; Annual Report Bulletin, Haiq, Ethiopia; 2014.

[16] Meadows LM, Morse JM, Morse JM, Swansen JM, Kuzel A (2001). Constructing evidence within the qualitative project. Nature of qualitative evidence.

[17] Creswell JW (2007). Qualitative inquiry and research design. Choosing among five traditions.

[18] Weiss RS (1994). Learning from strangers: the art and method of qualitative interview studies.

[19] Anderson R, Nichter M (1992). The efficacy of ethnomedicine: research methods in trouble. Anthropological approaches to the study of ethnomedicine.

[20] Sanders Thompson VL, Johnson-Jennings M, Baumann AA, Proctor E (2015). Use of culturally focused theoretical frameworks for adapting diabetes prevention programs: a qualitative review. Prev Chronic Dis.

[21] Peterson DR, Fox JP, Hall CE, Elveback LR (1970). The practice of epidemiology. Epidemiology: man and disease.

[22] Beattie J, Middleton J (1969). Spirit Mediumship and society in Africa.

[23] Ulin PR, Segall MH (1980). Traditional health care delivery in contemporary Africa.

[24] Antonitto A (1983). Traditional Medicine in Somalia; An Anthropological Approach to the concepts concerning Disease.

[25] Yehya NA, Dutta MJ (2010). Health, religion, and meaning: a culture-centered study of Druze women. Qual Health Res.

[26] Lidell C, Barrett L, Bydawell M (2005). Indigenous representations of illness and AIDS in Sub-Saharan Africa. Soc Sci Med.

[27] Atkinson S, Haran D (2005). Individual and district scale determinants of user’s satisfaction with primary health care in developing countries. Soc Sci Med.

[28] Kickbush I (2007). The move towards a new public health police. Ottawa 1986: The Fulcrum of Global Health Development. Promot Educ Suppl.

[29] Mishra SI, Hess J, Luce PH (2003). Predictors of indigenous healer use among Samoans. Altern Ther Health Med.

[30] Mesfin H, Teferi G, Heather B (2015). Traditional healing and primary care: a socio-cultural study in a rural *Tehuledere* community, North-Eastern Ethiopia. Ethiop J Health Dev.

[31] Federal Ministry of Health. Proceedings of the First National Conference on the Revision of National Health Policy. A.A. 2015.

[32] Braine T (2005). Health systems research is the best medicine. Bull World Health Organ.

