# Supplementary material for: Beliefs and perception of ill-health causation: a socio-cultural qualitative study in rural North-Eastern Ethiopia
Source: BMC Public Health. 2017 Jan 26;17:124. doi: 10.1186/s12889-017-4052-y (PMC5267452; doi:10.1186/s12889-017-4052-y)
Supplement: Additional file 1: — Focus Group Discussions and In-depth Interviews Guide. (DOCX 17 kb) [file 12889_2017_4052_MOESM1_ESM.docx]

**Additional file**

**Focus Group Discussions and In-depth Interviews Guide**

**(English Version)**

**(Beliefs and Perception of Ill-Health Causation: A Conceptual Understanding Based on a Socio-Cultural Study of the *Tehuledere* Community, North-Eastern Ethiopia)**

| To better understand participant’s experiences of ill-health causation, focus group discussion and in-depth personal interviews with adult community members will follow a semi-structured interview format. |
| --- |

**Note: *Encourage participants to tell the story in their own way and in their own words.***

**At start of interview, collect basic demographic information: age, length of residency in *Tehuledere,* level of education, living arrangements, marital status, and number of family members.**

1. I’m interested in knowing what it is like the health condition of your community. Can you tell me the major health problems commonly experienced by your families and village?
2. Could you explain your understanding of health? Illness?
3. What is your understanding of your family health?
4. Tell me about your families [specific condition] illness?
5. How would you describe illnesses and health in your family and village generally?

***Probe***: from the perspectives of natural and supernatural beliefs and perceptions;

***Probe:*** *Tell me* if you have other beliefs and perceptions of ill-health

***Probe:*** *Tell me more about* social elements of ill-health

***Probe:*** *If so, what are the main perceived differences? How is this expressed in local language? What are the local terms for ‘supernatural’; ‘natural’ and others (if any) explanations of ill-health’?*

1. What are the main causes of illnesses in your communities?

***Probe:*** What particular illnesses are believed to be caused through supernatural, natural and other (if any); please give me specific example?

***Probe:*** I am interested in knowing more about your experiences related to your beliefs related to supernatural, natural and other causes of illness. Could you describe your experiences when your parent became ill due to supernatural causes? Natural causes? Other causes of illness?

***Probes:*** *How is this expressed in local language? What are the local terms for ‘supernatural’; ‘natural’ and others (if any) causes of ill-health’?*

1. When a family member is diagnosed with illness ( supernatural, natural and social), a lot of things can change, not only in the life of the individual but also in the lives of others. How have things related to everyday activities, healthcare and life in general changed since your family was diagnosed with [specific illness]? Describe the changes for you and for your family.

**Probe:** focus on social causes of illness here

1. I am interested in knowing more about your experiences related to causes of illnesses and health care. How can indigenous beliefs and perceptions of ill-health be improved so that indigenous way of ill-health perceptions co exists with the biomedical one for the betterment of your health?

If there is something we discussed today that you want to discuss with me further or elaborate on, please feel free to contact me.
